# Protective Effects of High-Density Lipoprotein on Cancer Risk: Focus on Multiple Myeloma

**DOI:** 10.3390/biomedicines12030514

**Published:** 2024-02-24

**Authors:** Alessandro Allegra, Giuseppe Murdaca, Giuseppe Mirabile, Sebastiano Gangemi

**Affiliations:** 1 Hematology Unit, Department of Human Pathology in Adulthood and Childhood “Gaetano Barresi”, University of Messina, Via Consolare Valeria, 98125 Messina, Italy; aallegra@unime.it (A.A.); giuseppe.mirabile@polime.it (G.M.); 2 Department of Internal Medicine, University of Genova, Viale Benedetto XV, 16132 Genova, Italy; 3 Allergology and Clinical Immunology Unit, San Bartolomeo Hospital, 19038 Sarzana, Italy; 4 Allergy and Clinical Immunology Unit, Department of Clinical and Experimental Medicine, University of Messina, Via Consolare Valeria, 98125 Messina, Italy; gangemis@unime.it

**Keywords:** multiple myeloma, cancer, lipid metabolism, low-density lipoprotein, high-density lipoprotein, inflammation, oxidative stress, angiogenesis, prognosis

## Abstract

Lipid metabolism is intrinsically linked to tumorigenesis. And one of the most important characteristics of cancer is the modification of lipid metabolism and its correlation with oncogenic signaling pathways within the tumors. Because lipids function as signaling molecules, membrane structures, and energy sources, lipids are essential to the development of cancer. Above all, the proper immune response of tumor cells depends on the control of lipid metabolism. Changes in metabolism can modify systems that regulate carcinogenesis, such as inflammation, oxidative stress, and angiogenesis. The dependence of various malignancies on lipid metabolism varies. This review delves into the modifications to lipid metabolism that take place in cancer, specifically focusing on multiple myeloma. The review illustrates how changes in different lipid pathways impact the growth, survival, and drug-responsiveness of multiple myeloma cells, in addition to their interactions with other cells within the tumor microenvironment. The phenotype of malignant plasma cells can be affected by lipid vulnerabilities, and these findings offer a new avenue for understanding this process. Additionally, they identify novel druggable pathways that have a major bearing on multiple myeloma care.

## 1. Introduction

### 1.1. General Consideration on Lipid Metabolism

The second most prevalent hematologic cancer, multiple myeloma (MM), is characterized by the clonal proliferation of malignant plasma cells. The use of proteasome inhibitors, immunomodulatory medicines, anti-CD38 monoclonal antibodies, autologous transplants, and the B-cell maturation antigen targeting therapy has improved overall survival even though the condition is still mostly incurable. Regretfully, despite these developments, patients who continue to worsen following treatment have a low median survival rate [1]. Because of this, identifying new players in the beginning and development of the pathology is crucial to identifying new targets for treatment.

The interaction of the marrow microenvironment with malignant plasma cells was a significant discovery [2]. It is now widely recognized that the bone marrow microenvironment, which includes, among other cell types, bone marrow adipocytes, fibroblasts, stromal cells, osteoclasts, osteoblasts, and endothelial cells, influences the clinical course of MM, and each of these cell types plays a role in the pathogenesis and progression of the disease.

New data about this complicated environment points to a potential involvement for bone marrow adipocytes in the course of the disease. Dyslipidemia seems to be a newly identified prognostic marker for the course and fate of diseases, suggesting that the dysregulation of the lipoprotein transport system may be crucial to the appearance, prognosis, and even treatment of diseases. A number of studies have shown that changes in lipid metabolism and solid tumors are closely related.

The biogenesis of high-density lipoproteins (HDLs) occurs exclusively in systemic circulation with the participation of apolipoproteins, lipid transporters, such as ATP binding cassette A1 (ABCA1) and G1 (ABCG1), and the plasma enzyme, such as lecithin–cholesterol acyltransferase (LCAT). HDL metabolism also involves additional steps, in which the plasma enzyme cholesteryl ester transfer protein (CETP) mediates the exchange of cholesteryl ester (CE), present in HDLs, for triglycerides (TGs) present in triglyceride-rich lipoproteins (TRLs) (chylomicrons, chylomicron remnants, and very low-density lipoproteins [VLDLs]). This enzymatic exchange is a critical crossing point for all three lipoprotein pathways, where TGs from TRLs enter the HDL pathway, and CE from HDLs enter the chylomicron and VLDL pathways [3].

The chylomicron pathway, which is critical for the absorption and distribution of dietary lipids, and the VLDL/intermediate density lipoprotein (IDL)/low density lipoprotein (LDL) pathway, which is essential for the delivery of endogenously synthesized lipids from the liver to the peripheral tissues, and the HDL pathway, which is essential for the redistribution of peripheral cholesterol and other lipids among various tissues, are the metabolic pathways that are responsible for transporting lipids in circulation. Apolipoproteins, enzymes, lipid transfer proteins, and lipoprotein receptors are only a few of the many proteins that are involved in these pathways and support lipid homeostasis in general [4].

**Figure 1:**
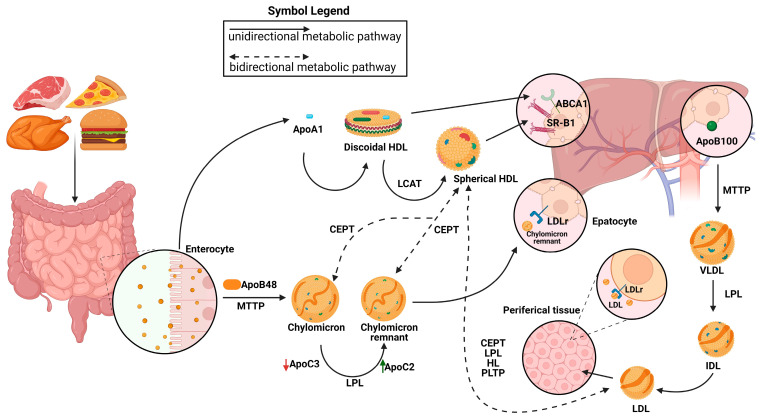
Representation of metabolic pathways of lipoprotein. Chylomicrons are synthesized from the intestine and are elaborated by the members of the low-density lipoprotein (LDL) receptors. Very low-density lipoprotein (VLDL)/intermediate density lipoprotein (IDL)/LDL particles are produced by the liver and the LDL clearance is due to the LDH receptors. The high-density lipoprotein (HDL) metabolic pathway involves the synthesis of discoidal HDLs and the formation of mature spherical HDLs. Their clearance is due to the action of the SR-BI receptor. The exchange of lipids between HDLs, chylomicrons, chylomicron remnants, and VLDLs is also represented. LPLs are essential for the conversion from chylomicron (CM) to CM remnants in the exogenous pathway and from VLDLs to LDLs in the endogenous pathway. Apolipoprotein C-II (APOC2) is a critical factor for LPL activity. Apolipoprotein C-III (APOC3) competes with LPL for binding to lipid emulsion particles. MTTP: microsomal triglyceride transfer protein; CEPT: cholesteryl ester transfer protein; LPL: lipoprotein lipase; PLTP: phospholipid transfer protein; ABCA1: ATP-binding cassette transporter; ApoB100: apolipoprotein B; ApoB48: apolipoprotein B48; LCAT: lecithin–cholesterol acyltransferase.

After lipids are loaded onto Apolipoprotein B48 (Apo B-48) molecules by the intestinal microsomal triglyceride transfer protein (MTTP), chylomicrons are formed within the enterocytes. Chylomicrons are produced and then secreted into the bloodstream via lymphatic circulation. There, they undergo conversion to chylomicron remnants by lipoprotein lipase (LPL) and acquire apolipoprotein E (ApoE), which facilitates their removal by low-density lipoprotein receptor (LDLR) superfamily members. Hepatic MTTP facilitates the transfer of hepatic lipids onto Apolipoprotein B100 (Apo B100) during VLDL assembly, resulting in the production of nascent VLDL particles that are subsequently released straight into systemic circulation. Like chylomicrons, VLDL triglycerides (TGs) are digested by plasma LPL, first transforming into IDL and then into LDL particles, which are subsequently eliminated from the bloodstream by the LDL receptor superfamily’s members. LDL, VLDL, and chylomicron remnants are eliminated from the bloodstream mainly by means of LDL receptor superfamily members (LDLR and LRP1) [5]. It has also been proposed that Heparan sulfate proteoglycans (HSPGs) are involved in this process, maybe by drawing lipoproteins that are in circulation onto cell membranes [6,7] and exposing them to LDL receptors [8], LRP1, VLDLR, and scavenger receptors. It has been demonstrated that HSPGs, primarily syndecan-1, functions as a separate receptor for TG-rich lipoprotein remains in the liver [9]. Furthermore, ApoE is assumed to facilitate the interaction between LPL and HSPGs, which reinforces the LPL group’s stability [10,11]. The ability of HSPGs to respond to important tumor microenvironmental stressors, such as hypoxia, by increasing the recruitment of HDL, LDL, and VLDL particles, is one of its new roles [12] (Figure 1).

### 1.2. HDL Metabolism and Cancer

The primary component of the cell membrane, lipids, are crucial for cell shape and proliferation [13], and previous research has suggested that changes in lipid metabolism may be linked to specific types of carcinogenesis [14], but there are contradicting findings in the literature.

Since the HDL is the lipoprotein that is most prevalent in most species, it is likely that this particle has significant roles. A rising body of research indicates that the HDL may have anti-inflammatory, anti-oxidative, and anti-apoptotic effects in addition to regulating innate and adaptive immune responses [15,16,17,18]. Low HDL levels may contribute to the development of cancer by disrupting some of these characteristics [18].

Variations in HDL cholesterol levels have been linked to an increased risk of many cancers, including colorectal, lung, and endometrial cancers [19,20,21,22,23,24,25,26,27,28,29,30]. Nevertheless, sometimes the findings have been inconsistent. Furthermore, it is unclear which protein component of the HDL, apolipoprotein A1, best describes the connection between the HDL and cancer risk [31].

However, according to a Mendelian randomization meta-analysis of the APOE gene, Asians had a 14% higher chance of developing cancer overall for every 1 mg/dL decrease in their genetically determined HDL-C level. According to prospective research, individuals in the highest HDL-C quintile among 27,074 male smokers in Finland had a 65% reduced risk of non-Hodgkin lymphoma (NHL) than those in the lowest quintile [22]. It is interesting to note that during the first ten years of follow-up, this inverse correlation was strongly found, but not during the subsequent follow-up. On the other hand, there was no significant correlation found between the risk of cancer and changes in total cholesterol, LDL-C, or triglyceride levels [20].

In another study, Knekt et al. demonstrated that men in the lowest quartile of total cholesterol had a five times higher risk of lymphoma or leukemia compared to those in the higher quintiles among non-smokers [32]. Nonetheless, a Mendelian randomization analysis revealed that, in contrast to genetically lowered LDL-C levels, low plasma LDL-C levels were robustly related with an increased risk of cancer [33]. These data may imply that low LDL-C levels do not cause cancer.

Lastly, a study that evaluated low plasma HDL cholesterol and apolipoprotein A1 as cancer risk factors examined participants from two population-based cohorts: the Copenhagen General Population Study (107,341 patients) and the Copenhagen City Heart Study (9387 patients) and followed them prospectively until the end of 2016 [34]. They saw 8748 malignancies in the Copenhagen General Population Study and 2164 in the Copenhagen City Heart Study throughout a 25-year follow-up period. Multivariable adjusted hazard ratios (HRs) for any cancer were 1.13 for people with HDL cholesterol levels of 58–77 mg/dL, 1.18 for people with HDL cholesterol levels of 39–58 mg/dL, and 1.29 for people with HDL cholesterol levels < 39 mg/dL in the Copenhagen General Population Study, when compared to people with HDL cholesterol levels ≥ 77 mg/dL. Accordingly, HRs for any cancer were 1.06 for those with apolipoprotein A1 levels of 160–189 mg/dL, 1.18 for those with apolipoprotein A1 levels of 130–159 mg/dL, and 1.28 for those with apolipoprotein A1 levels < 130 mg/dL, in contrast to those with apolipoprotein A1 levels > 190 mg/dL. Low levels of HDL cholesterol and/or apolipoprotein A1 were linked to a higher risk of breast cancer, lung cancer, nervous system cancer, non-Hodgkin lymphoma, and myeloproliferative neoplasm among twenty-seven different cancer types. The overall findings in the Copenhagen City Heart Study for men and women individually were comparable. Thus, there is a correlation between the elevated risk of many malignancies and HDL levels. The strongest increases in risk were seen in hematological and nervous system cancers, with smaller increases in breast and respiratory cancers.

### 1.3. HDL Metabolism and Hematological Malignancies

Data presented in the last experimentation [34] allow us to conclude that an important role appears to exist in the field of hematological neoplasms as well. Indeed, a recent study [35] verified the associations between HDL-C levels and the main categories of blood malignancies. Using data from the Korean National Health Insurance Service, a competing risks regression model was utilized to look at the hazard ratios of hematologic malignancies in 9,596,145 persons. The following hematologic tumors were measured for incidence: myeloid leukemia (ML), lymphoid leukemia (LL), NHL, and Hodgkin lymphoma (HL). In 79,179,225 persons who received follow-ups for 8.3 years on average, 15,864 incident hematologic malignancies were found. Subjects in the lowest HDL-C quartile had the highest risk of all hematologic cancers combined as well as of each specific kind of blood cancer, as comparison to those in the highest HDL-C quartile. A low HDL-C level was found to be linked to an elevated risk of hematologic malignancy, indicating that it is a preclinical marker and independent risk factor for hematologic malignancy. The authors have unequivocally shown that a low HDL-C level is linked to a higher risk of both general and specific forms of hematologic malignancies. This negative correlation held true for all study participant subgroups.

A further study that assessed the serial change in the lipid profile of 238 individuals who had hematologic tumors looked into a different facet of the relationship between lipids and hematological malignancies [36]. The HDL-C level in this group was lower than in the control group. Furthermore, compared to the control group, the patient group’s HDL-C level difference was more pronounced across all other lipid fractions. Interestingly, when patients experienced remission following treatment, the HDL-C level was reversed. The risk of NHL was found to be strongly correlated with the HDL-C level, but not with the total cholesterol or non-HDL-C level, according to the Alpha-Tocopherol Beta-Carotene Cancer Prevention Study [22]. HDL-C is the most sensitive measure of tumor burden, according to research conducted by Moschovi et al. on children with acute lymphoblastic leukemia [37]. After reaching remission, the abnormally low HDL-C values at the time of the leukemia diagnosis were restored. Alford et al. looked into the connection between lymphomagenesis and serial changes in cholesterol levels. They concluded that cholesterol levels drop years before a diagnosis of cancer [38].

Additional evidence supports the notion that lipid metabolism and hematological malignancies are closely related. When children were diagnosed with acute lymphoblastic leukemia, there was a reduction in Apo A-I levels [39]. On the other hand, patients with newly diagnosed CLL may also have hypocholesterolemia. Enhanced LDL clearance or enhanced cholesterol uptake by CLL cells appear to be the cause of hypocholesterolemia. Cholesterol levels may have an impact on the disease’s stage and activity [40].

## 2. Protective Effects of HDL on Neoplastic Diseases

The mechanisms underlying the reported findings could be related to the HDL’s potential immunomodulatory, anti-oxidative, and anti-apoptotic properties, which we will examine in more detail in the following sections. However, the HDL may also be able to regulate proliferative and inflammatory pathways in cancer development [41,42].

Through its interactions with lipid rafts on immune cell receptor-rich cellular membranes [43], the HDL may modify and prime the immune system to achieve a more advantageous anti-cancer state. The fact that stem and progenitor cell proliferation has been demonstrated to be inhibited by HDL cholesterol and apolipoprotein A1 is another intriguing feature [44,45].

Furthermore, according to additional mechanistic research, apolipoprotein A1 itself possesses anti-tumorigenic qualities through decreased angiogenesis, increased cholesterol efflux, and reverse cholesterol transport from cancer cells; these mechanisms may impede the growth or multiplication of tumor cells [46,47].

As previously mentioned, HDL-C itself may have anti-inflammatory and antioxidant qualities that help prevent cancer [17]. Cancer genesis and leukemogenesis are caused by a variety of processes, including immune-stimulating inflammatory pathways and genetic changes that impact oncogenes [48].

By reducing the proliferation rates of interleukin-3 and granulocyte-monocyte progenitors in bone marrow cells, HDL-C inhibits leukocytosis and myeloid proliferation [15]. Additionally, due to its anti-inflammatory properties, apolipoprotein A-I (Apo A-I), the main protein component of HDL-C, has preventive effects against the development of cancer [49].

As demonstrated by non-Hodgkin’s lymphoma [50], there is a crosstalk between oxidative stress, dyslipidemia, and low-grade chronic inflammation in hematological malignancies. Among the processes leading to carcinogenesis are inflammatory pathways triggered by immunological stimuli and genetic changes impacting oncogenes [51]. By reducing the growth of interleukin-3 and granulocyte-monocyte progenitors in bone marrow cells, HDL-C inhibits myeloid proliferation and leukocytosis [52]. When children with acute lymphoblastic leukemia were diagnosed, lower Apo A-I levels were noted [39].

On the other hand, decreased HDL-C levels could be a byproduct of cancer cell metabolism. To create new membrane biogenesis, malignant cells might stimulate liposynthesis and accumulate intracellular cholesteryl esters [53]. On the surfaces of tumor cells, there is a high expression of the HDL-C receptor scavenger receptor class B type (SR-BI). Significantly lower plasma HDL-C levels are the result of SR-BI’s facilitation of the absorption of cholesteryl esters from HDL-C into the cytoplasm [54]. Although specific mechanistic information is still lacking, it is known that the mTOR pathway is activated in multiple myeloma and may have a significant impact on this process by upregulating the SR-BI [55,56,57,58]. Preclinical research in the same setting revealed a correlation between low cholesterol levels in the culture media and the growth of neoplastic cells, indicating that cholesterol is utilized by lymphoma cells to advance [59,60].

Furthermore, hematologic malignancies like chronic lymphocytic leukemia have been linked to low cholesterol levels [40]. Additionally, studies have demonstrated that the successful treatment of leukemia and lymphoma can restore reduced HDL-C levels to normal, indicating that HDL-C may have a role as a biomarker of tumor burden [61,62]. Even after eight years, the low HDL-C group’s elevated cancer risk was still there, according to the cumulative incidence curves. Recent research has demonstrated that leukemogenic mutations may occur years before clinical leukemia diagnoses, which suggests that this finding may represent the latency of cancer [63].

Not to be overlooked is the well-established impact that obesity plays in the development of cancer. Obesity was identified by the International Agency for Research on Cancer as a “preventative factor” for MM. Regarding the effect of obesity on the results of hematopoietic cell transplantation, there is, however, contradictory data. While some studies have found no significant link, others have established a correlation between obesity and worse outcomes [64]. Increases in circulating quantities of cytokines, such as insulin-like growth factor-1, hepcidin, tumor necrosis factor-alpha, and interleukin-6, are linked to obesity and are thought to play a critical role in the pathophysiology of multiple myeloma [65,66].

Recent research has demonstrated the interaction between MM cells and bone marrow adipocytes (BMAs). BMAs, which are derived from non-hematopoietic BMSCs, have the properties of both brown and white adipose tissues and serve as an endocrine organ and energy storage [67]. In their bone marrow, patients with MM typically have higher numbers of pre-adipocytes and noticeably larger mature adipocytes [68]. It is interesting to note that BMAs stimulate autophagic proteins in plasma cells [69], which promote tumor growth and shield cancerous cells from chemotherapy-induced apoptosis [70]. The development of both MM and BMAs appears to be correlated with obesity, dyslipidemia, and metabolic syndrome. Furthermore, supporting the idea that obesity plays a significant role in the onset and progression of multiple sclerosis, it is well-known that disruption in the HDL metabolic pathway is linked to increased BMA deposition [71,72].

Compared to the overweight/obese group, those with a BMI < 25 kg/m^2^ showed a greater correlation between low HDL-C levels and hematologic cancers. Similar findings were made by a meta-analysis of cohort studies on the incidence of leukemia and obesity, which revealed that being overweight or obese was linked to an increased risk of leukemia, with a dose-dependent relative risk of 1.39 for overweight people and 1.39 for obese people [73]. All four of the major subtypes of leukemia showed a consistent increased risk of leukemia in the obese group. Chronic inflammation and weakened immunological function are linked to obesity [74], and hematologic malignancies are frequently associated with these conditions. Thus, in comparison to the non-obese group, the impact of low HDL-C levels on the incidence of hematologic malignancies may be less pronounced in the overweight/obese group.

Additionally, a study has suggested that Apo A1 may exert strong anti-tumorigenic effects on melanoma cells via a variety of mechanisms, including upregulating the expression of CD8 T cells, elevating anti-tumor macrophages, reducing angiogenesis, obstructing tumor growth, and minimizing tumor invasion and metastasis [47].

HDLs downregulate the generation of costimulatory molecules and antigen presentation by suppressing the synthesis of cytokines and chemokines in monocytes, macrophages, and monocyte-derived dendritic cells [75]. Furthermore, HDL–immune cell interaction can affect immune cell activation [76], although B-cell and T-cell receptors are localized in lipid rafts and are important for immune synapsis [77,78]. It looks like a fascinating area of research, even though there are not any current studies evaluating the impact of lipoproteins on the immune systems of myeloma patients or their potential therapeutic implications.

However, as Apo A1 is an acceptor of cholesterol at the first stage of ABCA1/ABCG1-mediated HDL production, the anti-tumorigenic action of Apo A1 may also be linked to ABCA1 and ABCG1 [79]. ABCA1, a significant cellular cholesterol efflux transporter, has been linked in a number of studies to potential roles in the etiology of cancer [80]. According to one study, promoter hypermethylation in prostate cancer causes ABCA1 downregulation, which raises intracellular cholesterol levels and creates an environment that is favorable for tumor growth [81].

Nevertheless, some research found that ABCA1/ABCG1 had a distinct involvement in the polarization of macrophages during tumor progression [15]. According to one study, the lack of ABCG1 prevented tumor growth by causing the macrophages within the tumor to change from tumor-promoting M2 to tumor-fighting M1 [82]. The Apo A1 mimetic peptide dramatically reduced the growth of tumors in mice models of breast cancer and ovarian cancer [83,84].

It is important to keep in mind, though, that decreased HDL-C levels could be a byproduct of cancer cell metabolism. Ras-mediated PI3-kinase activation leads to protein kinase B activation, which causes hypocholesterolemia by inhibiting cholesterol efflux and inducing LDL receptor-mediated cholesterol influx, thereby maintaining a high intracellular cholesterol level for cell proliferation [85]. To create new membrane biogenesis, malignant cells can stimulate liposynthesis and intracellular cholesteryl ester buildup. On the surfaces of tumor cells, there is a high expression of the HDL-C receptor SR-BI. This receptor lowers the amount of HDL-C in plasma by facilitating the absorption of cholesteryl esters from HDL-C into the cytoplasm [54]. Moreover, aggressive tumors and unfavorable clinical results were linked to elevated SR-BI expression [86].

## 3. HDL and Multiple Myeloma

According to certain research, low HDL levels are also more common in patients with other hematologic malignancies, like MM, which may be an early indicator of illness [34,35].

Numerous investigations have demonstrated that individuals with MM had significantly decreased levels of total cholesterol (TC), HDL-C, and LDL-C [87,88,89]. Nonetheless, the relationship between lipid profile and subsequent cancer risk has only been examined in a small number of research studies. A recent Danish study involving 117 thousand people found that low levels of HDL-C cholesterol were significantly associated with an increased risk of MM (aHR (95% CI) for one standard deviation (SD) decrease in HDL-C = 1.73 (1.28–2.35)) [34]. Similarly, a study conducted as part of the Women’s Health Initiative, which involved twenty-four thousand women, found a borderline inverse association between HDL-C and MM (adjusted hazard ratio (aHR), 95% confidence interval (95% CI) for highest to lowest quartile = 0.56, 0.31–1.01) [90]. Low HDL levels were linked to an increased risk of hematologic malignancy, including MM (aHR, 95% CI for lowest to highest quartile = 1.63, 1.48–1.78), according to a different study [35].

Furthermore, even day-to-day, lipid levels can vary significantly over time [91,92]. Lipid variability is now recognized as a separate characteristic from the actual lipid content [93]. According to recent epidemiologic data, variations in lipid levels from visit to visit are linked to a number of health outcomes, including mortality, end-stage renal disease, coronary heart disease, and stroke [94,95,96,97,98]. A recent population study conducted in Korea discovered that people with greater fluctuations in HDL levels and lower baseline HDL levels were more likely to acquire MM. These results bolster the hypothesis that dysregulated lipid metabolism poses a risk for MM [99].

A crucial part of the HDL, ApoA, is currently thought to play several advantageous roles in cancer prevention in addition to atherosclerosis, thrombosis, and diabetes [100]. According to a systematic review, patients with MM appear to have decreased levels of HDL-C in comparison to controls. Furthermore, with respect to the remaining lipid profile markers, apoA-I showed the biggest decreases, with TC following closely behind. While inter-disease variations were noted, similar results were previously obtained by studies evaluating cancer patients generally [101]. Numerous large-scale population-based investigations have demonstrated that low cholesterol is linked to a higher likelihood of MM existence; however, these abnormalities may resolve if remission is achieved [61,62].

The most likely “answer” to both genetic and environmental alterations is the metabolome, which is the last result of a biological process and, hence, the most representational of the phenotype [102].

According to a study, individuals with varying MM International Staging System (ISS) stages had distinct metabolomic profiles [103]. When ISS I patients were compared to ISS III patients, the profile showed higher levels of leucine, phospholipids, high-density lipoproteins, low-density lipoproteins, apolipoproteins A1 and A2, and cholesterol. Patients with IgA and IgG paraproteins had different metabolomic profiles, mostly because IgA patients had larger levels of high- and low-density lipoprotein subfractions [103].

Finally, 502,507 people from the UK Biobank were included in a major prospective cohort research study that was followed up to 2019 and evaluated for TC, LDL, HDL, and ApoA and ApoB values as risk factors for plasma cell neoplasms [104]. In order to ascertain if cholesterol levels had a causal relationship with the development of plasma cell neoplasms, they also employed two-sample Mendelian randomization. They followed up on 1819 cases of plasma cell neoplasm in the UK Biobank over 14.2 years. Researchers discovered that a decreased incidence of plasma cell neoplasm was connected with higher baseline blood serum cholesterol levels. Triglycerides did not show an inverse relationship with the incidence of plasma cell neoplasm, but all other lipid profiles examined in this study did. Genetically predicted serum levels of LDL, HDL, and total cholesterol did not, however, appear to be associated with multiple myeloma. Thus, there was a correlation shown to increase the incidence of plasma cell neoplasm with low serum levels of total cholesterol, LDL, HDL, ApoA, and ApoB.

### 3.1. HDL Effects in Multiple Myeloma

**Figure 2:**
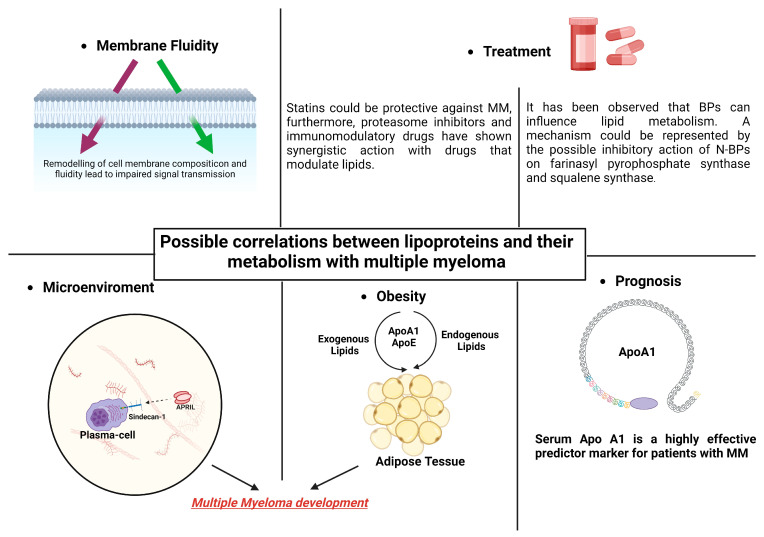
Lipid modifications that can alter the fluidity of MM cells’ membranes may be the cause of alterations in intracellular signaling. The development of MM and obesity may be influenced by lipoprotein’s actions on adipocytes. Combining statins and bisphosphonates with MM therapy may have synergistic effects. In individuals with MM, apolipoprotein might be significant in terms of prognosis.

However, it is unclear how exactly MM patients’ low lipid levels are caused to manifest, and it has yet to be determined if changes in metabolism and the beginning of gammopathy are causally related. As we shall see in the next paragraphs, the effects of HDLs in myeloma disease probably occur at various levels. Some of the ways that HDLs work include through their impacts on cellular fluidity, their impact on the medullary microenvironment, and their effects on obesity and concomitant dyslipidemia. The association between dyslipidemia and hematological pathology is confirmed by the relationship shown between anti-dyslipidemic medications and MM and between anti-myeloma therapies and HDLs, as will be shown later (Figure 2).

First of all, cholesterol may be used by malignant cells. In general, cholesterol is crucial for the formation and function of cell membranes in both normal and malignant cells [105], and the structure and functionality of the cell membrane may be a potential mechanism that, at least partially, mediates the effects of the lipid and lipoprotein transport system in the development of MM.

The extracellular stimulants of cell division and proliferation primarily target the cell membrane and its protein constituents. Fluidity is a crucial physical characteristic of cell membranes and is essential to their biological activity [106]. Changes in the cholesterol content of the cell membrane have been shown to have an effect on membrane fluidity [107], which can then have an effect on the structure, antigenicity, and responsiveness of proteins on the cell surface, such as G-coupled receptors and ion channels, to external stimuli. For instance, a reduced erythrocyte plasma membrane lipid content is associated with an increased activity of erythrocyte Na^+^/Li^+^ countertransport, Na^+^/K^+^ cotransport, and Na^+^/K^+^ pump activity [108]. Consequently, this could have an impact on biological reactions and intracellular signaling, like in the instance of pancreatic B-islets secreting insulin. The physical characteristics of the membrane can be altered by lipoproteins, which can also assist in regulating the activity of transmembrane proteins and the motor-dependent elongation of interior organelles, including the endoplasmic reticulum [109,110,111,112].

Several studies have established the existence of basic connections between inflammation and MM oxidative stress [113,114,115,116]. It has also been discovered that the HDL, by its anti-inflammatory and antioxidative qualities, regulates inflammatory pathways in the progression of cancer [117]. The HDL and its ApoA may cause immune cells’ lipid rafts and free cholesterol levels to drop, which would in turn lessen signaling pathways that promote cell proliferation and inflammation [52]. ApoA’s antitumorigenic properties were well illustrated in an in vivo investigation. Through indirect changes to macrophage and other immune cell function, the mechanism by which this happens may have anticancer consequences [47]. Furthermore, via HDL-related pathways, SR-BI is a key player in the control of the quiescence, differentiation, and proliferation of hematopoietic stem and progenitor cells. Moreover, compared to other cell types, the expression of SR-BI is noticeably higher on the surfaces of tumor cells. HDL cholesteryl esters can be taken up by SR-BI and absorbed into the cytoplasm, which lowers plasma HDL levels significantly [44,54]. Therefore, a premalignant state may potentially be the cause of the low HDL level.

Elevated levels of oxidized LDL cholesterol are correlated with a low concentration of HDL cholesterol. Consequently, increased oxidative stress levels inside cells have been connected to the pathophysiology of cancer [118,119]. Therefore, a low quantity of LDLs may result in less ox-LDLs and less damage to long-lived plasma cells from oxidation.

Recent in vitro research, however, suggests that exogenous cholesterol is a necessary metabolite for the survival of myeloma cells. However, the enhanced absorption of LDLs by myeloma cells may account for the low LDL plasma levels seen in MM patients [120]. On the other hand, low levels of HDL, LDL, and total cholesterol may be the cause of cancer growth.

Like in solid tumors, the relationship between lipoproteins and obesity may also have a value in the case of MM. There is mounting evidence that the lipid and lipoprotein transport systems, which regulate endogenously created and exogenously obtained lipids through diet, are key players in the development of morbid obesity. Diet-induced obesity is also largely influenced by a number of other factors, including the most prevalent protein component of HDLs, APOA1, and ApoE, a protein component of HDLs, LDLs, and VLDLs, and the functional ligand of LDLR in the removal TRLs from circulation [121,122,123,124,125,126,127,128]. Thus, it is conceivable that APOA1 and ApoE have an impact on the mechanisms linked to the onset and advancement of MM.

Furthermore, syndecan-1 has been demonstrated to be a receptor for TG-rich lipoprotein remnants in the liver, as previously described. MM cells have a surface protein called syndecan-1, which is essential for their interaction with the bone marrow microenvironment [129,130,131,132,133]. High levels of cell surface syndecan-1 expression are a hallmark of myeloma tumors, with the HS chains of this protein being crucial for MM cell proliferation and survival in the BM microenvironment [134]. Additionally, it has been discovered that syndecan-1 functions as APRIL’s co-receptor [135]. APRIL is a crucial component of the BM microenvironment and promotes the survival of MM cells. Notably, myeloma growth in vivo is stimulated by the soluble form of syndecan-1 [136]. Furthermore, MM cell proliferation was inhibited, and the rate of apoptosis increased when syndecan-1 was suppressed [137].

### 3.2. Prognostic Value of HDL in Multiple Myeloma Patients

In MM, HDLs can potentially serve as a stand-in prognostic marker [138]. In Zhongshan Hospital in Shanghai, China, 307 MM patients were enrolled retrospectively between 2007 and 2016 [139]. The pre-diagnostic serum lipid profile’s predictive importance was assessed by the authors. Using the prognostic factors that were found, a new Lasso Cox regression model was built. The results demonstrated a substantial change in lipid levels between the ISS stages: the late ISS stage had reduced levels of LDL, cholesterol, and Apo A1 and B. But in terms of cause-specific survival (CSS), progression-free survival (PFS), and overall survival (OS), only Apo A1 demonstrated statistical relevance. Longer OS was seen in those with greater Apo A1 levels. Moreover, a multivariate analysis showed that Apo A1 was an independent predictor. Compared to the Durie and Salmon (DS) system and the International Staging System (ISS), the Zhongshan Score model demonstrated superior accuracy. In conclusion, serum Apo A1 is a highly effective predictive marker for patients with MM out of all the serum lipid profiles. The prognosis of MM patients, lipid content and ratios, and treatment responses are correlated, which implies that lipid profiles may have clinical implications for judgment and decision-making.

Lastly, a study sought to investigate a predictive risk-stratification model combining lipid profiles and clinical characteristics in patients with multiple myeloma [140]. 275 patients’ data were retrospectively analyzed, and the training and validation cohorts were split up at random. A univariate and multivariate Cox analysis was used to identify the prognostic markers, which included HDL, TG, LDL, Apo B, and Apo A1 ratios, and lactate dehydrogenase. The construction of a six-factor prognostic model was based on a Lasso regression model. Following patient classification into low- and high-risk groups, the former group exhibited a longer OS duration. The risk score model’s area under the curve (AUC) for the five- and ten-year OS was 0.756 and 0.940, respectively, showing higher accuracy than the DS stage and the ISS.

To create a prognostic model, this study integrated the lipid metabolic profile with the clinical features of individuals with MM. The prediction accuracy was improved by the nomogram that integrated the risk score and ISS stage. This model can monitor the lipid profile as an easy-to-use and practical tool, which has some clinical importance for enhancing prognostic accuracy and identifying targets for treatment.

### 3.3. Future Perspectives and Conclusions

In the future, MM might be prevented and treated with the knowledge gained regarding the connections between lipid metabolism and myeloma disease. For example, there is increasing evidence that statin use helps prevent MM [141,142,143]. The liver produces less cholesterol when statins, which are competitive inhibitors of HMG-CoA reductase, are taken. When hepatic cholesterol synthesis is inhibited, the endoplasmic reticulum’s cholesterol levels drop. This causes the sterol regulatory element binding proteins (SREBPs) to migrate from the endoplasmic reticulum to the Golgi, where proteases cleave them into active transcription factors. The LDL receptor and HMG-CoA reductase are two of the most significant genes that are expressed more when the SREBPs translocate to the nucleus [144]. The increased expression of the LDL receptor causes an increase in the number of LDL receptors on the plasma membrane of hepatocytes, which accelerates the clearance of lipoproteins that contain apolipoprotein B and E (LDL and VLDL), while the increased expression of HMG-CoA reductase returns hepatic cholesterol synthesis to normal [144]. Triglyceride and plasma LDL-C levels have decreased, which can be explained by the enhanced clearance of LDLs and VLDLs. Some studies have demonstrated that statins decrease the generation and release of VLDL particles by the liver in addition to decreasing LDL and VLDL levels via speeding up the clearance of lipoproteins. Statins raise HDL-C levels, but it is unclear how they do so [145].

Due to their potential anticancer effects, statins have received a significant increase in interest in recent years. It appears that statins affect either the upregulation or inhibition of important pathways related to cancer, including the reduction of cancer stemness and the prevention of angiogenesis, metastasis, and proliferation. Moreover, it has been discovered that statins cause cancer cells to undergo oxidative stress, cell cycle arrest, autophagy, and death. It is interesting to note that clinical research has linked statin use to a lower risk of cancer development, a lower cancer grade at diagnosis, a lower chance of local recurrence, and an increased patient survival rate [146,147,148].

Over 240 studies are underway on the possibility of intervening with statins on neoplastic disease and at least five with the aim of intervening on MM (Table 1).

Numerous data deserve to be reported. Low HDL-C levels are a component of the metabolic syndrome, which is associated with MM [149]. The well-known “HDL hypothesis” has long linked low HDL-C levels to cardiovascular diseases (CVD) [150]. Cardiovascular risk is higher in MM patients than in the general population [151,152,153,154]. Additionally, dyslipidemia associated with MM may not respond to conventional lipid-lowering treatments, and statins may be protective against MM [149]. Furthermore, proteasome inhibitors and immunomodulatory medications have shown a synergistic effect with lipid-modulating medicines.

Other drugs used in patients with MM seem to confirm the relationship between lipid metabolism and gammopathy. Bisphosphonates have a key function in the management of myeloma bone damage and work in concert with antimyeloma medications [155,156], and nitrogen-containing bisphosphonates (N-BPs) are commonly used to treat tumor-associated osteolysis because they prevent osteoclast-mediated bone resorption. Numerous mechanisms have been suggested, such as direct cytotoxic or cytostatic effects on tumor cells, antiangiogenic actions, the inhibition of osteoclastogenesis and osteoclast-mediated bone resorption, and the prevention of tumor cell invasion of bone [157,158]. Furthermore, zoledronic acid (ZA) can improve dendritic cells’ capacity to boost innate and adaptive immunity, and BPs have immunomodulatory effects that are mediated by the buildup of mevalonate metabolites in tumor cells, which activate T lymphocytes expressing the T-cell receptor [159,160].

It has been established that BPs can affect lipidic metabolism, even though their exact mode of action is yet unknown. N-BPs can inhibit farnesyl pyrophosphate synthase and squalene synthase. A study assessed patients with smoldering myeloma at diagnosis to investigate the impact of ZA on serum lipids [161]. ZA was administered to sixteen individuals at baseline as well as in months 1, 2, 4, and 6. The controls were the other ten patients. TGs, C-terminal telopeptide of type I collagen (CTX), HDL-C, LDL-C, and TC levels were evaluated at baseline and after 1, 3, and 6 months in all subjects. The authors noted a progressive and considerable reduction in TC in treated patients, with a maximum decrease of 13% after 6 months. In addition, LDL-C levels dropped by 21% after six months, although TGs and HDL-C levels showed no discernible changes. Additionally, following ZA administration, the cardiovascular risk indices showed improvement: the TC/HDL-C ratio gradually dropped by 17%, while the HDL-C/LDL-C ratio climbed by 36%, suggesting a cumulative effect. In conclusion, ZA administered intravenously at large dosages appears to be able to improve the atherosclerosis risk index while also changing the lipid profile in individuals with smoldering myeloma.

N-BPs have also been shown to have a significant effect on lipid metabolism in patients with Paget’s bone disease receiving pamidronate every three months or in osteoporotic women receiving repeated neridronate infusions [162,163]. Even while 90% of this biosynthesis was restored after 48 h, in vitro experiments using prostatic cell lines indicate that ZA can suppress the formation of cholesterol [164].

In the future, more drugs with the ability to affect lipid metabolism may be investigated in relation to malignancies and MM. Proprotein convertase subtilisin/kexin type 9 (PCSK9), for example, has developed into a novel therapeutic target for hypercholesterolemia and related cardiovascular illnesses, as well as a crucial enzyme in lipid metabolism. PCSK9 has abnormal expression in a variety of cancer types and plays a critical role in both carcinogenesis and cancer immunity [165].

A major advancement in the treatment of changes in lipid metabolism has been made with the introduction and clinical approval of novel PCSK9 inhibitory therapies, which include three monoclonal antibodies (Tafolecimab, Evolocumab, and Alirocumab) and one small interfering RNA (Inclisiran). These therapies may also be investigated in relation to neoplastic diseases [166,167].

Finally, the research on the connections between lipid metabolism and the circadian clock is particularly intriguing, partly because of the potential therapeutic ramifications. The circadian clock is a regulatory mechanism, which is made up of regulated and clock genes, that controls an organism’s physiological and metabolic activity in a rhythmic manner and synchronizes it with environmental changes. The metabolic rhythm is primarily regulated by core transcription genes and the downstream regulatory genes that result from them. These genes control the synthesis and breakdown of proteins. Numerous studies looked at the relationship between the circadian rhythm and cancer in relation to lipid metabolism regulation. Targeting the important fatty acid synthesis-related enzymes SREBP, ACLY, ACC, FASN, and SCD has been suggested as a viable cancer treatment approach. It could be able to stop tumor growth by interfering with lipid metabolism by blocking these enzymes [168,169].

This implies a close relationship between the circadian clock and lipid production. Therefore, in addition to taking into account the timing and duration of drug administration, effective combination therapy should target lipid production. In the end, tailored chronotherapy can improve medication effectiveness in the management of cancer and accomplish treatment objectives [170].

Lastly, further research might be conducted on other HDL action mediators in MM. Vitamin D may have a particularly important role. Cholesterol is the main component of vitamin D and is where it originates [171]. Insufficient levels of vitamin D are connected to a number of illnesses, including dyslipidemia, which is defined by low levels of HDL. Vitamin D is also necessary for immunological regulation and bone metabolism. Due to HDL’s suppression during vitamin D deficiency, previous studies have linked low HDL levels to this condition [172,173]. A vitamin D deficit is frequently associated with an advanced stage of MM, an increased risk of progression, the development of pathological fractures, and a poorer prognosis [174]. Additionally, there is growing evidence, derived from both in vitro research and clinical trials, that vitamin D supplementation may be advantageous for patients with multiple myeloma as an adjuvant treatment. This is especially true when combined with cutting-edge chemotherapeutic agents like lenalidomide, bortezomib, and anti-CD38 antibodies [175].

In conclusion, there is hope for practical applications in comprehending the carcinogenic pathways of lipoprotein metabolism and focusing on lipid metabolism reprogramming to find novel targets for MM treatment. It is likely plausible to speculate that activating escape metabolic pathways may be a more effective method to suppress MM development than focusing only on restricting the lipid pathway. Thus, in the near future, the coordinated inhibition of several lipid metabolism-related pathways should be investigated in relation to preclinical and clinical MM research.

## Figures and Tables

**Table 1:** Ongoing studies on the use of statins in MM (www.clinicaltrial.com entry 13 January 2024).

Study Title	Condition	Interventions	Study Type	Phase	Status	NCT Number
Simvastatin as Inhibitor of Cell Adhesion Mediated Drug Resistance in Patients With Refractory Multiple Myeloma	Multiple Myeloma	Drug: Simvastatin	Interventional	Phase 2	Completed	NCT00399867
The Effect of High Dose Simvastatine on Multiple Myeloma	Multiple Myeloma	Drug: Simvastatin	Interventional	Phase 1 Phase 2	Completed	NCT00281476
Donor Atorvastatin Treatment in Preventing Severe Acute GVHD After Nonmyeloablative Peripheral Blood Stem Cell Transplant in Patients With Hematological Malignancies	Multiple Myeloma and 21 more Hematological Malignancies	Drug: Atorvastatin, Calcium	Interventional	Phase 2	Completed (Has results)	NCT01527045
Overcoming Chemotherapy Resistance In Refractory Multiple Myeloma With Simvastatin and Zoledronic Acid	Multiple Myeloma	Drug: Simvastatin and zoledronic acid	Interventional	Not applicable	Terminated (Has results)	NCT01772719
Atorvastatin in myeloma	Multiple myeloma	Drug: atorvastatin	Interventional	Phase 1	Unknown	NCT00164086

## Data Availability

Not applicable.
